# Effectiveness of eHealth interventions in long-term follow-up care for breast and gynecological cancer survivors: a systematic review

**DOI:** 10.1007/s00520-026-10781-0

**Published:** 2026-05-26

**Authors:** Nicole Barbara Luzia Kuhn, Lisa-Marie Maukel, Ana N. Tibubos

**Affiliations:** 1 Department Nursing Science, Diagnostics in Healthcare & eHealth, University of Trier, 54286 Trier, Germany; 2 Department of Psychosomatic Medicine and Psychotherapy, University Medical Centre of the Johannes Gutenberg-University Mainz, Untere Zahlbacher Str. 8, 55131 Mainz, Germany

**Keywords:** Breast cancer, Gynecological cancer, Delivery mode, Long-term follow-up care, Outcome measures, Effectiveness of eHealth interventions

## Abstract

**Purpose:**

As survival rates for breast and gynecological cancers improve, many survivors experience persistent long-term challenges, including fatigue, psychological distress, and reduced health-related quality of life (HRQoL). eHealth interventions may offer a scalable and accessible approach to support survivorship care. This systematic review aimed to evaluate the effectiveness of eHealth interventions in long-term follow-up care for breast and gynecological cancer survivors.

**Methods:**

A systematic literature search was conducted across six databases (PubMed, PsycINFO, Web of Science, Cochrane Library, MedRxiv, and PsyArXiv) for studies published between January 2010 and February 2026. Eligible studies included adult breast or gynecological cancer survivors and evaluated eHealth interventions in follow-up care compared with usual care. Outcomes included outcomes such as burden of treatment, HRQoL, self-management, hospital readmissions, complications, infections, fatigue, or malnutrition. Data were extracted, synthesized narratively, and methodological quality was assessed using established risk-of-bias tools.

**Results:**

A total of 41 studies were included, predominantly involving breast cancer survivors, with very limited representation of gynecological cancer populations. Overall, methodological quality ranged from low to moderate, with higher risk of bias observed in nonrandomized studies. eHealth interventions were associated with small to moderate improvements in fatigue, anxiety, depression, HRQoL, and treatment adherence, particularly among breast cancer survivors. However, effect sizes were generally smaller in studies with lower risk of bias. Evidence for gynecological cancer survivors was scarce and inconclusive, with only one study exclusively focusing on this population. Long-term outcomes were rarely assessed, and no studies evaluated the defined outcomes infections, malnutrition and unplanned hospital admissions.

**Conclusions:**

eHealth interventions show potential to support long-term survivorship care, particularly in improving psychological outcomes and quality of life among breast cancer survivors. However, the overall evidence base is limited by methodological heterogeneity, short follow-up periods, and the underrepresentation of gynecological cancer populations. Further high-quality, long-term, and cancer-specific research is needed to better establish the effectiveness and generalizability of these interventions in survivorship care.

**Implications for cancer survivors:**

As survivorship care needs continue to increase, eHealth interventions may contribute to more accessible, scalable, and patient-centered models of follow-up care. However, their long-term effectiveness and applicability across diverse cancer populations remain unclear. More rigorous, long-term, and cancer-specific research is required to better define their role in survivorship care.

## Introduction

The global burden of cancer is increasing, with an estimated 28.4 million new cases projected by 2040 [1]. Breast and gynecological cancers account for a substantial proportion of this burden. In 2020, breast cancer alone contributed 2.3 million new cases and 685,000 deaths globally, with incidence expected to exceed 3 million cases by 2040 [2, 3]. Gynecological cancers—including ovarian, cervical, endometrial, vulvar, and vaginal cancers—accounted for approximately 1.47 million new cases worldwide in 2022 [3, 4]. At the same time, cancer-related healthcare expenditures continue to rise, with costs in the USA projected to increase from $183 billion in 2015 to $246 billion by 2030 [5]. Together, these trends highlight the growing demand for sustainable long-term cancer care. Advances in early detection and treatment have substantially improved survival. In the USA, 5-year relative survival for breast cancer increased from 75% in 1975 to 91% in 2020 and for ovarian cancer from 36 to 51% [6, 7], with similar trends observed in Europe [8, 9]. However, improved survival has not been matched by equivalent progress in survivorship care.

Survivorship is frequently associated with persistent physical and psychosocial challenges, including fatigue, anxiety, depression, and reduced health-related quality of life (HRQoL) [10]. Breast and gynecological cancer survivors report unmet needs, particularly in relation to psychological support, symptom management, and rehabilitation [11–17]. Structured and systematic assessment of these needs is rarely implemented in routine care [18], and both populations express a strong desire for more psychological support and structured follow-up care [11, 14–19]. This reflects a broader gap between increasing care needs and the capacity of healthcare systems to provide coordinated, long-term follow-up [19–24]. These issues can further impair functioning and well-being for years after treatment ends [20–24], underscoring the need for accessible, sustainable, and effective survivorship care.

At the same time, healthcare systems are under pressure to manage rising numbers of survivors with limited resources and unequal access to follow-up care. eHealth interventions—including mobile health (mHealth) applications, telemedicine, and digital self-management platforms—have been proposed as a strategy to address these challenges by enabling scalable, accessible, and patient-centered care [25, 26]. Existing evidence suggests that eHealth interventions may improve selected outcomes such as symptom management and emotional well-being [27–30]. Most studies focus on short-term or treatment-phase outcomes, with limited evaluation of sustained effects in the post-treatment period. Far less is known about their effectiveness in the long-term follow-up phase, particularly for breast and gynecological cancer survivors. Previous research has primarily focused on short-term outcomes and specific intervention components [31–43].

Patients’ support needs emerge predominantly in the follow-up phase [44]. Survivors expressed positive attitudes toward digital interventions, especially those incorporating activity tracking, personalized feedback, and information on the effects of exercise on mood and fatigue [44, 45]. However, usability studies have identified several limitations, including challenges related to platform navigation, mobile compatibility, and the degree of personalization [46, 47]. Despite their potential, eHealth interventions are not yet widely integrated into standard care. Cancer-related applications score poorly in quality assessments [48], and most are developed by commercial providers without input from patients or clinicians [49].

Taken together, these gaps highlight the need for a comprehensive synthesis of evidence on eHealth interventions in long-term follow-up cancer care. Understanding which modalities are most effective, which outcomes can be improved, and how these interventions are best delivered is critical for informing future survivorship care models [50]. Given this gap, this systematic review aims to evaluate the effectiveness of eHealth interventions in long-term follow-up care for breast and gynecological cancer survivors. The review was guided by the following research questions:

1. In women with breast or gynecological cancer who have completed initial treatment, what are the effects of eHealth follow-up interventions, compared with standard care, on clinical outcomes such as treatment burden, health-related quality of life (HRQoL), self-management, hospital readmissions, complications, infections, fatigue, and malnutrition?
2. What are the key components, content, and modes of delivery of these eHealth follow-up interventions?

## Methods

The review protocol was developed using the Preferred Reporting Items for Systematic Review and Meta-Analyses (PRISMA) guidelines [51] and registered with the International Prospective Register of Systematic Reviews (PROSPERO; registration number CRD42023392467). A comprehensive literature search was conducted across four databases (PubMed, PsycINFO, Web of Science, and Cochrane Library) and two preprint platforms (MedRxiv and PsyArXiv) to identify relevant studies published between January 2010 and February 2026. Searches were conducted independently by two authors (NK, LLM) in February 2026.

The search strategy combined terms related to the population (e.g., breast and gynecological cancers), intervention (e.g., eHealth, mHealth, telehealth), follow-up care, and outcomes (e.g., burden of treatment, HRQoL). Boolean operators and truncation techniques were applied to optimize the search. Two independent reviewers (NK, LMM) screened titles, abstracts, and full texts of potentially relevant studies. Disagreements were resolved through discussion or consultation with a third reviewer (ANT).

### Inclusion and exclusion criteria

A study was considered eligible for inclusion in the systematic review if

1. it included adult breast cancer or gynecological cancer survivors,
2. it evaluated eHealth interventions (e.g., telehealth, mobile applications, or web-based platforms),
3. it included a comparator (e.g., usual care),
4. it reported outcomes such as burden of treatment, HRQoL, self-management, hospital readmissions, complications, infections, fatigue, or malnutrition,
5. and it used an interventional study design (randomized controlled trial (RCT), nonrandomized controlled trial (non-RCT), cluster randomized trial, cohort study, case control study, controlled before after study).

In this review, gynecological cancers refer to malignancies of the female reproductive system, including ovarian, cervical, endometrial, vulvar, and vaginal cancers. These entities were represented in the search strategy using both general terms (e.g., “female genital neoplasms”) and specific diagnostic terms (e.g., ovarian and uterine cancers).

A study was excluded if

1. it focused on prevention, treatment, initiation, or palliative care,
2. it was not peer-reviewed,
3. it was a descriptive study with quantitative outcomes, literature review, or noninterventional study.

The search was limited to studies published between January 2010 and February 2026.

Two reviewers (NK, LLM) independently extracted data using a standardized form. Disagreements were resolved through discussion with a third reviewer (ANT). Full-text review was then conducted by two reviewers (NK, LMM). The following information was extracted from the included studies: author, year, country, study design, sample size and population characteristics, eHealth intervention content and delivery mode, comparator, outcome measures and measurement tools, duration and follow-up, effectiveness of the eHealth intervention, key findings, and key intervention content areas (Table 1).

**Table 1:** Characteristics and key findings of included studies

Ref no.	Author (year) *Country*	Design/type of study	Sample size Type of study population	eHealth intervention Content Delivery mode	Comparator	Outcome measures and measurement tools	Duration and follow-ups	Effectiveness of eHealth intervention Key findings	eHealth intervention content Key areas
[52]	Bahar-Bandani et al. (2022) *Iran*	Randomized controlled trial	*n* = 38 Breast cancer	Mobile health educational intervention *n* = 17 Mobile health (mHealth) application	Control group did not receive any messages *n* = 17	Fatigue *(cancer fatigue scale (CFS))* Body image *(body image concern inventory scale (BICI))*	7 weeks	The mean score of cancer fatigue scale after the intervention in the intervention group was decreased significantly (*p* = 0.005), but no statistically significant difference was observed in the control group. There was a *significant difference* in the mean score of *body image* concern inventory in the intervention group (*p* = 0.002) after the intervention compared with the control group.	Education and self-management
[53]	Børøsund et al. (2020) *Norway*	Randomized controlled trial Baseline 3-month follow-up	*n* = 172 Mixed cancer types (population included 48% breast cancer)	App-based stress-management intervention (StressProffen) *n* = 84 Mobile health (mHealth) application	Usual care control group *n* = 88	Primary outcome Perceived stress *(Perceived Stress Scale (PSS-14))* Secondary outcome Anxiety and depression *(Hospital Anxiety and Depression Scale (HADS))* Health-related quality of life *(36-Item Short Form Health Survey (Rand-36 version))* System use, usefulness, and ease of use *(post-intervention survey for acceptability and feasibility)*	3 months	*Decreased stress* (mean difference [MD] − 2.8; 95% confidence interval [CI], [− 5.2 to − 0.4]; *p* = 0.022) and improved *HRQoL *(role physical MD = 17.7, [CI 3.7–31.3], *p* = 0.013; social functioning MD = 8.5, [CI 0.7–16.2], *p* = 0.034; role emotional MD = 19.5, [CI 3.7–35.2], *p* = 0.016; mental health MD = 6.7, [CI 1.7–11.6], *p* =.009). *No significant changes were observed for anxiety or depression.*	Psychosocial support and mental health
[54]	Børøsund et al. (2022) *Norway*	12-month randomized, controlled trial	*n* = 172 Mixed cancer types (population included 48% breast cancer)	StressProffen, a digital application (app)–based stress management intervention for cancer survivors *n* = 84 Mobile health (mHealth) application	Usual care *n* = 88	Primary outcomes Perceived stress *(Perceived Stress Scale (PSS-14))* Secondary outcome Anxiety and depression *(Hospital Anxiety and Depression Scale (HADS))* Self-regulatory fatigue *(Self-Regulatory Fatigue 18 (SRF-18))* Health-related quality of life (HRQOL) *(36-Item Short Form Health Survey (Rand-36 version))*	12 months	Over the 12-month study time, the intervention group reported *significantly decreased stress *(*p* < 0.001), *depression* (*p* = 0.003), and *self-regulatory fatigue* (*p* = 0.002) as well as *improved HRQOL* (for 6 of 8 domains, *p* ≤ 0.015) in comparison with controls. The largest favored effects for the intervention group were observed at 6 months: *stress* (estimated mean difference [MD], −5.1; *p* < 0.001), *anxiety* (MD, −1.4; *p* = 0.015), *depression* (MD, −2.1; *p* < 0.001), *self- regulatory fatigue* (MD, −4.9; *p* < 0.001), and *HRQOL* (7 of 8 domains; *p* ≤ 0.037).	Psychosocial support and mental health
[55]	Chow et al. (2024) *Hong Kong*	Pilot randomized controlled trial	*n* = 35 Breast cancer (55.6%) Gynecological cancer (44,4%)	App-based education and nurse counseling, Women’s Wellness After Cancer Program (WWACP) app *n* = 18 Mobile health (mHealth) application	Attention from research nurse *n* = 17	Feasibility *(recruitment rate, consent rate, retention rate, counselling session attendance rate, percentage of modules accessed, and number of days active)* Intervention outcomes Sense of coherence *(Chinese version of the Sense of Coherence 13-item Scale (CSOC-13))* Cancer-specific distress *(Chinese version of the Impact of Events-Revised Scale (CIES-R))* Health-related quality of life *(Hong Kong Chinese version of the MOS 36-item) (Short Form Health Survey version-2.0 (SF-36v2))* Acceptability *(individual semistructured interviews)*	12 weeks	The intervention participants reported to have *significant improvement in physical well-being* at T1 (Cohen’s *d* effect size (*d*) = 1.04, 95% CI 0.24, 1.83), *sense of coherence* (*d* = 0.76, 95% CI − 0.03, 1.54), and *cancer-specific distress* (*d* = 1.03, 95% CI − 1.83, − 0.21) at T2.	Psychosocial support and mental health
[56]	Cohen et al. (2019) *USA*	Three‐armed randomized controlled feasibility trial	*n* = 40 Breast cancer	Aerobic exercise and technology guided mindfulness training Exercise and relaxation training *n* = 13 Virtual reality (VR) and interactive tools	Aerobic only *n* = 14 Relaxation only *n* = 13 Aerobic	Feasibility and acceptance (*study satisfaction questionnaires)* Fatigue *(12-item, Piper Fatigue Scale (PFS))* Tiredness and energy *(Activation Deactivation Adjective Check List (AD ACL) subscales (Energy and Tiredness))* Anxiety and depression *(Hospital Anxiety and Depression Scale (HADS))*	Not reported	More *favorable postintervention evaluations* were reported by the *combined group*, compared to aerobic exercise or relaxation only (*p* < 0.05). Reductions in fatigue favoring the combined group (*p* = 0.05) showed a modest effect size (Cohen's *d* = 0.91) compared to aerobic exercise only.	Physical activity and exercise
[57]	Ferrigno-Guajardo et al. (2025) *Mexico*	Randomized controlled trial	*n* = 68 Breast cancer	Online MBSR intervention *n* = 33 Web-based program Mobile health (mHealth) application	Waitlist control group *n* = 35	Primary outcomes Anxiety *(Generalized Anxiety Disorder-7 (GAD-7) scale)* Depression *(Patient Health Questionnaire-9 (PHQ-9))* Fatigue *(Functional Assessment of Chronic Illness Therapy-Fatigue-Brief (FACIT-F))* Insomnia *(Insomnia Severity Index (ISI))* Cancer-related worry *(Cancer Worry Scale (CWS))* Vasomotor symptoms *(subdomain of the Menopause-Specific Quality of Life questionnaire (MENQOL))* Mindfulness and awareness *Mindfulness practice (Mindful Attention Awareness Scale (MAAS))*	Postintervention, 5 and 8 months	Compared to the control group, MBSR participants showed *significant reductions in anxiety *(mean difference −4.13 points, 95% CI −6.79 to −1.46, *p* = 0.003), *depression* (mean score difference −6.03, *p* < 0.001), *fatigue* (mean difference + 6.03, *p* = 0.002), *insomnia* (mean difference −3.97, *p* = 0.026), and *cancer-related worry* (mean difference −4.57, *p* = 0.003) at postintervention, but *no change in vasomotor symptoms* (*p* > 0.05). MBSR participants also demonstrated *increased mindful awareness* (mean difference + 1.00, *p* = 0.004) that *persisted through follow-up*. The proportion of participants with clinically significant anxiety decreased from 96% pre-intervention to 38% at 8-month follow-up in the MBSR group, compared to relatively stable rates (88 to 87%) in the control group.	Psychosocial support and mental health
[58]	Freeman et al. (2015) *USA*	Multisite randomized trial	*n* = 118 Breast cancer	Telemedicine delivery of an imagery-based behavioral intervention Live delivery (LD), *n* = 48 Telemedicine delivery (TD), *n* = 23 Telehealth services	Waitlist *n* = 47	General health-related quality of life *(Medical Outcomes Study 36-item short form survey (SF-36))* Breast cancer–specific quality of life *(Functional Assessment of Cancer Therapy-Breast (FACT-B))* Perceived cognitive function *(Functional Assessment of Cancer Therapy-Cog (37-item FACT-Cog (version 2))* Spiritual well-being *(23-item Functional Assessment of Chronic Illness Therapy (FACIT))* *Spiritual Well-Being Expanded Scale (Functional Assessment of Chronic Illness Therapy (FACIT-Sp-EX; version 4))* Psychological distress *(18-item Brief Symptom Inventory (BSI-18))* *Sleep disturbances (9-item Pittsburgh Sleep Quality Index (PSQI))*	6 weeks postintervention, 3 months	The Bonferroni method was used to correct for multiple comparisons, and alpha was adjusted to 0.01. LMM analyses revealed *less fatigue, cognitive dysfunction, and sleep disturbance* for LD and TD compared to WL across the follow-up (*p*’s < 0.01). Changes in fatigue, cognitive dysfunction, sleep disturbance, and health-related and breast cancer-related QOL were clinically significant. There were no differences between LD and TD.	Psychosocial support and mental health
[59]	Galiano-Castillo et al. (2016) *Spain*	Randomized controlled trial	*n* = 81 Breast cancer	Internet-based exercise program, telerehabilitation e-CUIDATE *n* = 40 Telehealth services	Usual care, control group *n* = 41	Quality of life *(European Organization for the Research and Treatment of Cancer Quality of Life Questionnaire (Spanish version of the EORTC QLQ-C30 and its BC))* Pain *(Brief Pain Inventory short form (BPI))* Isometric handgrip strength (Takei Scientific Instruments Co. (*TKK 5101 Grip-D; Takey, Tokyo, Japan))* Isometric abdominal strength Isometric back strength (Takei Scientific Instruments Co. *TKK 5002 Back-A; Takey, Tokyo, Japan))* Lower body strength Fatigue *(Piper Fatigue Scale-revised (R-PFS))*	Postintervention, 8 weeks	Significantly improved scores for *global health status, physical, role, cognitive functioning, and arm symptoms *(all *p* < 0.01) as well as *pain severity* (*p* = 0.001) and *pain interference* (p = 0.045) compared with the control group. Significant improvements also were observed favoring the telerehabilitation group for *affected and nonaffected side handgrip* (both *p* = 0.006), *abdominal, back, and lower body strength* (all *p* < 0.01), and total fatigue (*p* < 0.001).	Physical activity and exercise
[60]	Graetz et al. (2018) *USA*	Randomized controlled feasibility trial	*n* = 44 Breast cancer	Web-based app that provided patients with the ability to report symptoms and AI medication use, with built-in alerts sent to a patient’s care team Mobile health (mHealth) application	“App” group, who were provided access to the app but did not receive weekly reminders	Primary outcome Aromatase inhibitor (AI) adherence *(Morisky Medication Adherence Scale (MMAS-4))* Secondary outcome Symptom burden *(Functional Assessment of Cancer Therapy-Endocrine Symptoms (FACT-ES))* App usage logs Intervention feedback *(telephone interview)*	Postintervention, 8 weeks	Participants in the App and Reminder group had higher weekly app usage rate (74% vs. 38%, *p* < 0.05) during the intervention and reported *higher AI adherence* at 8 weeks (100% vs. 72%, *p* < 0.05). Symptom burden increase was higher for the app group compared to the app + reminder group but did not reach statistical significance.	Health monitoring and symptom tracking
[61]	Horsbøl et al. (2025) *Denmark*	Randomized controlled trial	*n* = 288 Breast cancer	MyHealth follow-up program Digital symptom monitoring and self-management support, nurse-lead Telehealth services	Standard follow-up visits	Work ability *(Work Ability Score (WAS))*	6, 12, 24, and 36 months	Work ability increased significantly in both groups during the first 6 months (mean WAS increase MyHealth: 1.64, 95% confidence interval [CI]: 1.26, 2.02; control: 1.57, 95% CI 1.17, 1.97) and continued to increase slightly but nonsignificantly (*p* values > 0.13) until end of follow-up at 36 months. Improvement was especially pronounced among patients reporting poor work ability at baseline. *Differences in mean WAS between patients in MyHealth and control follow-up were nonsignificant* and close to zero at all time points (–0.21 to 0.48).	Education and self-management
[62]	Im et al. (2023) *USA*	Randomized controlled trial	*n* = 199 Breast cancer	Cultural tailored virtual information and coaching/support program (TICAA) Web-based platform *n* = 104	American Cancer Society (ACS) website *n* = 95	Primary outcome Supportive care needs (Supportive Care Needs Survey-Short form 34 (SCNS score)) Secondary outcomes Symptom distress (Memorial Symptom Assessment Scale Short Form (MSAS-SF)) Quality of life (Functional Assessment of Cancer Therapy (FACT-B scores))	Postintervention 4 and 12 weeks	The intervention group showed *significant reductions in their needs* from the baseline (T0) to post 4 weeks (T1) and to post 12 weeks (T2). Although the changes were not statistically significant, the intervention group had decreased symptoms from T0 to T2 while the control group had an increase in their symptoms. The intervention group had a *significant increase in their quality of life* from T0 to T2.	Education and self-management
[63]	Jacobs et al. (2022) *USA*	Randomized, controlled trial	*n* = 92 Breast cancer	Telehealth intervention (STRIDE) for patients taking AET after breast cancer *n* = 46 Telehealth services	Usual care *n* = 46	Satisfaction *(Client Satisfaction Questionnaire (CSQ-3))* Medication adherence *(Medication Adherence Report Scale (MARS-5))* Therapy satisfaction (*Cancer Therapy Satisfaction Questionnaire (CTSQ))* Symptom distress *(Breast Cancer Prevention Trial Symptom Scale (BCPT))* Quality of life (QOL) *(Functional Assessment of Cancer Therapy-Breast Cancer (FACT-B))* Anxiety and depression (*Hospital Anxiety and Depression Scale (HADS))* Stress coping *(Measure of Current Status (MOCS-A))* Self-efficacy *(Managing AET Symptoms Questionnaire (SESM-AET))*	Baseline, 12 weeks	Compared to MedMon, STRIDE patients reported *less symptom distress* (*b*[difference] = − 1.91; 95% CI [− 3.29, − 0.52], *p* = 0.007) and *better self-management of AET symptoms, coping, QOL, and mood.* No significant differences in AET satisfaction or adherence.	Health monitoring and symptom tracking
[64]	Kim et al. (2025b) *South Korea*	Randomized controlled trial	*n* = 49 Breast cancer	Smartphone-based self-management program BeHealth app *n* = 24 Mobile health (mHealth) application	Treatment as usual *n* = 25	Self-efficacy and health behavior *(Korean translation of a scale developed by Schwarzer and Renner)* Eating habits *(dietary habit and food-intake-frequency questionnaire developed by Oh *et al*.)* Physical activity *(Korean translation of the Godin Leisure-Time Exercise Questionnaire)* Selected cardiometabolic risk factors *(blood pressure, BMI, waist circumference, fasting blood glucose level)*	Baseline, 12 weeks	The experimental group showed *significantly increased levels of vegetable intake* (*p* = 0.017) and *significantly reduced levels of fasting blood glucose* (*p* = 0.037) compared to the control group, suggesting that the Be-Health app may be effective in reducing cardiovascular disease risk factors.	Education and self-management
[65]	Little et al. (2025) *UK*	Randomized controlled trial	*n* = 2712 Mixed cancer types (population included 52,2% breast cancer)	Digital self-management program Live Well, *n* = 906 Complex digital intervention Renewed, *n* = 903 Addressing symptom management, physical activity, diet, weight loss, and distress Renewed with support, *n* = 903 Renewed with additional brief support by email and telephone Web-based platform	Generic online advice *n* = 806	Primary outcomes Quality of life *(European Organisation for Research and Treatment of Cancer Quality of Life Questionnaire–Core 30 (EORTC-QLC-30 instrument (version 3) summary Score))* Secondary outcomes Quality of life *(European Organisation for Research and Treatment of Cancer Quality of Life Questionnaire (EORTC QLQ-C30 subscales), global self-rated health, symptom subscales, functional subscales)* Depression and anxiety *(fear of relapse and the Measure Yourself Concerns and Wellbeing questionnaire (MYCaW)) (modified enablement scale)* *Resource use (medication/consultation costs in primary care)* *Website usage*	Baseline, 6 and 12 months	*At 6 months, all groups improved* (primary time point: *n* for the generic, Renewed groups, and Renewed with support were 806, 749, and 705, respectively), with *no significant between-group differences for EORTC QLQ-C30, but global health improved more in both the Renewed groups*. By 12 months, there were *small improvements in EORTC QLQ-C30 for Renewed with support* (versus generic advice: 1.42, 95% confidence interval [CI] = 0.33 to 2.51); both Renewed groups improved global health (12 months: Renewed: 3.06, 95% CI = 1.39 to 4.74; Renewed with support: 2.78, 95% CI = 1.08 to 4.48), dyspnea, constipation and enablement, and *lower primary care NHS costs* (in comparison with generic advice [£265]: Renewed was −£141 [95% CI = −£153 to −£128] and Renewed with support was −£77 [95% CI = −£90 to −£65]); and for *Renewed with support improvement in several other symptom subscales, no harms were identified.*	Education and self-management
[66]	Lopez et al. (2023) *USA*	Pilot study	*n* = 35 Mixed cancer types (population included 60% breast cancer)	Smartphone-based application (APP) for delivery and monitoring of meditation *n* = 17 Mobile health (mHealth) application	Waitlist *n* = 18	Symptom burden *(Edmonton Symptom Assessment Scale (ESAS-FS))* Anxiety and depression *(Hospital Anxiety and Depression Scale (HADS))* Sleep quality *(Pittsburgh Sleep Quality Index (PSQI))*	Baseline, 2 weeks	Mixed model analysis revealed a statistically significant association between meditation length (5, 10, or 15 min) and change in anxiety, with 15-min sessions associated with greater reductions in anxiety. In the exit survey, more meditation group versus control group participants reported improved focus, mood, and sleep. Study groups differed significantly by ESAS fatigue score change; the meditation group decreased a median of 1.5 pts (IQR 2.5) and the control group increased a median of 0.5 points (IQR 2). The meditation group, but not the control group, *experienced statistically significant improvement in ESAS fatigue, depression, anxiety, appetite, and physical, psychological, and global distress*. Change in PSQI and HADS anxiety and depression scores did not reveal any statistically significant between-group differences.	Psychosocial support and mental health
[67]	Lozano-Lozano et al. (2022) *Spain*	Assessor-blinded randomized controlled clinical trial	*n* = 80 Breast cancer	BENECA mHealth app, 8-week intervention and a face-to-face occupational therapy program Mobile health (mHealth) application	Usual care group that only received the mHealth lifestyle app	Cognitive performance *(trail making test (TMT)) (Wechsler adult intelligence scale (WAIS))* Psychological state *(Hospital Anxiety and Depression Scale (HADS))* Pain perception (*Brief Pain Inventory (BPI))* Fatigue *(Piper Fatigue Scale (PFS))* Functional capacity *(6 min walk test (6MWT))*	Baseline, 8 weeks and 6 months	*Selective attention* (TMT) was significantly higher in the IA group, with a moderate to large effect size for TMT A (T2: *d* = 1.1; T 3: *d* = 1.2), *working memory and processing speed* (WAIS), *anxiety and general HADS score* (*d* = 1.6), and *functional capacity* at 8 weeks and 6 months (*d* = 1.5). *Fatigue perception* (mean difference, −0.6; 95% CI −1.4 to 0.04; *p* = 0.009) and *pain* (intensity level *p* < 0.001; interference level *p* = 0.002) were also significantly more improved in the IA group.	Psychosocial support and mental health
[68]	Okuyama et al. (2024) *Japan*	Randomized controlled trial	*n* = 125 Breast cancer	Symptom monitoring using ePRO app (Welby My Carte ONC) *n* = 61 Mobile health application (app)	Usual care *n* = 64	Health-related quality of life *(Japanese version of the Functional Assessment of Cancer Therapy-Breast (FACT-B) ver.4.0)* Communication between healthcare providers and patients *(European Organization for Research and Treatment of Cancer Quality of Life Questionnaire-COMU26 (EORTC QLQ-COMU26))* Patient-reported outcome *(Common Terminology Criteria for Adverse Events PRO‑CTCAE)* Medication adherence	3 months	In the ONC group, the response rate to PRO-CTCAE was about 70% or higher until week 10. The item missing rate was 0. The ONC group *reported more symptoms related to joint pain and insomnia*. The difference in *FACT-B *total score between the groups was − 1.55 (95% confidence interval: − 5.91, 2.81), *indicating no significant difference.*	Health monitoring and symptom tracking
[69]	Oswald et al (2022) *USA*	Randomized controlled trial	*n* = 30 Breast cancer	eHealth cognitive behavioral therapy intervention (CBT-I) for Spanish-speaking breast cancer survivors *n* = 15 Telehealth services	Waitlist control, participants received no intervention until after the posttreatment survey, opportunity to participate in the eHealth CBT-I intervention *n* = 15	Acceptability and feasibility *(Treatment Perceptions Questionnaire (TPQ))* Sleep quality (*7-item Insomnia Severity Index (ISI), 9-item Pittsburg, Sleep Quality Index (PSQI))*	6 weeks	Satisfaction with CBT-I was acceptable. Post-intervention, there were medium to large group differences for *average insomnia symptoms *(*d* = 1.02), *sleep disturbance* (*d* = 1.25), and *sleep efficiency* (d = 0.77) favoring CBT-I. There were small/medium to medium/large group differences for the proportion of participants with *clinically significant insomnia symptoms* (*d* = 0.52), *sleep disturbance* (*d* = 0.67), and low *sleep efficiency* (*d* = 0.33) favoring CBT-I. Spanish-language eHealth CBT-I for BCS was acceptable and feasible and showed preliminary efficacy. Spanish-language eHealth cognitive behavioral therapy intervention (CBT-I) showed large effects on insomnia symptoms and sleep efficiency. Very small sample (*n* = 30), preliminary evidence	Psychosocial support and mental health
[70]	Reeves et al. (2021) *Australia*	Randomized controlled trial	*n* = 159 Breast cancer	Remotely delivered, weight loss intervention with optional text messages *n* = 79 Telehealth services	Usual care *n* = 80	Primary outcomes Weight (*Tanita BWB-600 Weederburn Scales, Sydney, Australia)* Secondary outcomes Body composition *(total fat and lean mass)* Biomarkers of metabolic syndrome (*risk score, waist circumference, triglycerides, high density lipoprotein (HDL) cholesterol, systolic and diastolic blood pressure, fasting plasma glucose)* Quality of life *(Patient-Reported Outcome Measurement Information System (PROMIS) Global Health Scale)* Fatigue *(Functional Assessment of chronic Illness Therapy Fatigue Scale (FACIT))* Arthralgia *(Musculoskeletal Pain subscale, Breast Cancer Prevention Trial Symptom Scale (BCPT))* Menopausal symptom *(Greene Climacteric Scale (GCS))* Fear of cancer recurrence (*Concerns About Recurrence Questionnaire (CARS))* Body image *(Body Image and Relationship Scale (BIRS))* Adverse events *(self-reported adverse events categorized according to the Common Terminology Criteria for Adverse Events (CTC-AE, v4.0))*	Baseline, 6 months, 12 months, and 18 months	*Significantly greater weight loss* was observed in the intervention versus usual care arms at 12 months (− 4.5% [95% CI − 6.5, − 2.5], *p* < 0.001), which was largely maintained at 18 months (− 3.1% [− 5.3, − 0.9], *p* = 0.007). *Significant intervention effects on fat mass *were observed at each assessment, with greater loss of lean mass observed in the intervention versus usual care at all follow-up assessments, being statistically significant at 6 and 18 months. The intervention arm demonstrated *statistically significant and more favorable metabolic syndrome risk scores* across all follow-up assessments, which were statistically significant compared to usual care. Significant intervention effects favoring intervention were seen at 12 months for *physical quality of life* (*d* = 0.40), *musculoskeletal pain* (*d* = − 0.49), and *body image* (*d* = − 0.31), with nonsignificant, small (*d*≈0.2–0.3) improvements observed for mental quality of life and psychological menopausal symptoms. 25 serious AEs were observed (intervention: *n* = 13, usual care: *n* = 12) Long-term follow-up (12–18 months), many significant results (weight, fatigue, HRQoL, metabolism), RCT with adequate sample size	Physical activity and exercise
[71]	Seib et al. (2022) *Australia*	Multicenter, single-blinded, randomized controlled	*n* = 351 Mixed cancer types (population included 94.7% breast cancer, 2.8% gynecological cancer)	eHealth-enabled platform 12-week lifestyle intervention and virtual health consultation *n* = 175 Telehealth services	Standard care *n* = 176	Health-related quality of life (HRQoL) *(Functional Assessment of Cancer Therapy-General (FACT-G)) (Short Form Health Survey (SF-36))*	Baseline, 12 weeks	Following the 12‑week lifestyle program, intervention group participants reported statistically *significant improvements* in *general health, bodily pain, vitality, and global physical and mental health scores*. Improvements were also noted in the control group across several HRQoL domains, though the magnitude of change was less.	Psychosocial support and mental health
[72]	Solk et al. (2023) *USA*	Randomized full factorial trial	*n* = 266 Breast cancer	Fit2Thrive intervention (Fitbit and Fit2Thrive smartphone app) Mobile health (mHealth) application	None	Physical activity *(Moderate to Vigorous Physical Activity (MVPA physical activity measure))* Patient-reported physical and psychological health outcomes (PROs) *Patient-reported outcomes measurement information system (PROMIS)*	Baseline, 12 weeks and 24 weeks	*All PROMIS measures* except sleep disturbance *significantly improved* (*p*’s < 0.008 for all) from baseline to 12 weeks. Effects were maintained at 24-weeks. The “on” level of each component did not result in significantly greater improvements on any PROMIS measure compared to the “off” level.	Physical activity and exercise
[73]	Svendsen et al. (2025) *Norway*	Randomized controlled trial	*n* = 430 Breast cancer	Digital stress-management interventions (CBT-based and mindfulness-based) Digital cognitive behavioral therapy stress-management intervention CBI *n* = 140 Digital mindfulness-based stress-management intervention MBI *n* = 143 Mobile health (mHealth) application	Usual care *n* = 147	Primary outcomes Perceived stress (Perceived Stress Scale) Secondary outcomes Anxiety and depression *(Patient Health Questionnaire for Depression and Anxiety (PHQ4))* Fatigue *(Chalder’s Fatigue Scale)* Health-related quality of life *(RAND Corporation 36-Item Short Form Health Survey (RAND-36))* Mindfulness *(Baer’s 5 Facet Mindfulness Questionnaire (FFMQ-15))* Coping *(Theoretically Originated Measure of the Cognitive Activation Theory of Stress)* Sleep *(Norwegian Shift Work, Sleep and Health survey)* App use *(app progress/activity, time spent using the app, days from first to last use, number of completed module*)	6 months	Perceived stress level at baseline was low for all groups. *No statistically significant mean differences (MD) were detected between either of the intervention groups and the control group from baseline to 6-month follow-up for perceived stress level *(MBI: MD −0.28 [95% CI −1.75, 1.19], CBI: MD −0.42 [95% CI −1.89, 1.06]), nor for the majority of the secondary outcomes. After 6 months of access, the CBI and MBI stress-management interventions did not yield significantly improved outcomes for women with breast cancer compared with usual care controls.	Psychosocial support and mental health
[74]	Van der Hout et al. (2020) *Netherlands*	Nonblinded, randomized, controlled trial	*n* = 625 Mixed cancer types (population included 45% breast cancer)	Web-based eHealth application Oncokompas, (fully automated behavioral intervention) *n* = 320 Mobile health (mHealth) application	Usual care, access to Oncokompas after 6 months *n* = 305	Primary outcome Patient activation (*knowledge, skills, and confidence for self-management)* Secondary outcome Health-related quality of life (HRQOL) (*Quality of life questionnaire core 30 items (QLQ-C30))* Mental adjustment to cancer Supportive care needs Self-efficacy Personal control Perceived efficacy in patient-physician interaction	1 week, 3 months, 6 months	*Patient activation was not significantly different* between intervention and control groups over time (difference at 6-month follow-up 1·7 [95% CI − 0·8–4·1], *p* = 0.41). There were no significant effects on the secondary outcome measures. Mental adjustment to cancer, supportive care needs, self-efficacy, personal control or perceived efficacy in patient-physician interaction.	Education and self-management
[75]	Wu et al. (2025) Australia	Pilot randomized controlled trial	*n* = 55 Gynecological cancer	Online intervention for fear of recurrence, iConquerFear *n* = 29 Web-based platform	Waitlist control *n* = 26	Primary outcomes Feasibility (*iConquerFear uptake (i.e., proportion of women who accessed iConquerFear) and engagement (i.e., proportion of women completing the modules, number of logins, time spent) according to referral and website logs))* Acceptability *(Internet Evaluation and Utility Questionnaire (IEUQ))* Safety *(Distress Thermometer)* Secondary outcomes *(semistructured interviews were conducted to explore participants’ experiences using iConquerFear)* Exploratory outcomes Fear of cancer recurrence *(Fear of Cancer Recurrence Inventory-Short Form (FCRI-SF))* Fear of progression *(Fear of Progression Questionnaire- Short Form (FoP-Q-SF))*	Baseline, 8 weeks	Of 62 eligible survivors, 55 (61%) were randomized (intervention *n* = 29; control *n* = 26). At baseline, 55% (30/55) reported severe FCR (FCRI-SF ≥ 22). Of those randomized, 51% (*n* = 28) accessed iConquerFear; 16/28 (57%) users completed ≥ 3/5 modules. *Mean post-intervention acceptability score* (IEUQ) was 3/4 (SD = 0.8). Three (11%) users withdrew due to distress from iConquerFear. *Qualitative interviews (n = *13*) identified 6 key themes* (e.g., participant factors influencing engagement). *Differences between intervention and control group changes in FCR/P were nonsignificant.*	Psychosocial support and mental health
[76]	Zachariae et al. (2018) *Denmark*	Randomized controlled trial	*n* = 255 Breast cancer	Internet-delivered cognitive-behavioral therapy CBT-I *n* = 133 Web-based platform	Waitlist control *n* = 122	Sleep quality *(Pittsburgh Sleep Quality Index (PSQI))* Online version of the consensus sleep diary Fatigue *(Functional Assessment of Chronic Illness Therapy for Fatigue (FACIT-F))*	9 weeks and 6 weeks	*Statistically significant* (*p* ≤ 0.02) time group interactions were found for all *sleep-related outcomes* from pre- to postintervention. Changes in the proportion of nights on which participants took sleep medication did not reach statistical significance (*p* = 0.09). *Large effect sizes were found for improvements in insomnia severity *(ISI), *sleep quality* (PSQI), and *sleep efficiency.* Medium effect sizes for increased total sleep time, less time in bed, and fewer EMAS; and small effect sizes for shorter SOL, fewer NAs, reductions in fatigue (FACIT-F), and less time spend awake after sleep onset (WASO).	Psychosocial support and mental health
[77]	Zion et al. (2023) *Spain*	Double-blind randomized controlled trial	*n* = 449 Mixed cancer types (population included 52.8% breast cancer, gynecological cancer 8.0%)	App-based version of cognitive behavioral stress management (CBSM) Cognitive behavioral digital therapeutic *n* = 226 (Attune) Mobile health application (app)	Health education Sham app on anxiety and depression symptoms *n* = 223 (Cerena)	Primary outcome Anxiety (PROMIS-Anxiety Short Form, PROMIS-A) Secondary outcome Depression (PROMIS-Depression Short Form, PROMIS-D) Global impressions of change in anxiety and depression (Patient Global Impression of Change Scale (CGI))	4 weeks, 8 weeks, and 12 weeks	Patients randomly assigned to digitized CBSM showed *significantly greater reductions in anxiety *(*b* = −0.03; *p* = 0.019) and *depression* (*b* = −0.02; *p* = 0.042) symptoms over 12 weeks. Patients who received digitized CBSM were also significantly more likely to perceive much or very much improvement (v no/minimal change or much/very much worse) in their *symptoms of anxiety* (*p* < 0.001) and *depression* (*p* < 0.001) compared with the control.	Psychosocial support and mental health
[78]	Aref et al. (2023) *Iran*	Quasiexperimental pretest–posttest with control group	*n* = 140 Breast cancer	Mobile phone–based self-care education and counseling via video, text, and audio sessions *n* = 70 Mobile health (mHealth) application	Usual care *n* = 70	Primary outcomes General health *(General Health Questionnaire (GHQ-12))* Quality of life *(Breast Cancer General Quality of Life Questionnaire (QLQ-C30)) (Specific Quality of Life Questionnaire (BR23))* Secondary outcomes Symptoms *(Edmonton Symptom Assessment Scale (ESAS))*	6 weeks	*Difference in general health was significant* − 1.55 (95% CI − 2.29, − 0.80, *p* < 0.001), *but it was not significant in Edmonton symptoms* − 0.25 (95% CI − 5.85, 5.34, *p* = 0.166). Also, the *difference in general quality of life was significant* − 2.39 (95% CI − 2.46, 1.23, *p* = 0.018), and the *difference in specific quality of life was significant* − 4.31 (95% CI − 5.68, − 2.93, *p* < 0.001).	Education and self-management
[79]	Cairo et al. (2020) *USA*	Nonrandomized 2-group control study design	*n* = 127 Breast cancer	Mobile wellness coaching app with tailored individual and emotional support (Vida app) *n* = 66 Mobile health (mHealth) application	Self-group received the same printed survivorship information *n* = 61	Weight gain/loss *(body mass index (BMI))* Physical activity *(Godin Shephard Leisure-Time Physical Activity Questionnaire (GSLTPAQ))* Nutrition *(“Rate Your Plate” nutritional assessment (RYP))* Fatigue *(Visual Analog Scale (VAS))* Depression *(Patient Health Questionnaire (PHQ-2))*	6 months	At 6 months, more patients in the app group experienced *weight loss* and had a *significantly greater reduction in overall body mass index *(*p* < 0.01). The app group also demonstrated statistically *significant improvements *in *“strenuous” physical activity *(*p* = 0.04) and had *significant improvement *in their *dietary patterns* (*p* < 0.001), compared to the self-guided group. The app group had greater reduction in fatigue and improvement in depression, but these changes were not statistically significant.	Physical activity and exercise
[80]	Chan et al. (2024) *Hong Kong*	Feasibility study, prospective, single-arm study	*n* = 50 Breast cancer	Mobile app–based rehabilitation program called “THRIVE” Digital rehabilitation program including exercise tracking, medication reminders, and self-care support Mobile health (mHealth) application	None	Primary outcomes Feasibility and adherence Secondary outcomes Physical activity *(International Physical Activity Questionnaire (MVPA) short version)* Health-related quality of life *(European Organisation for Research and Treatment of Cancer Quality of Life Questionnaire (EORTC-QLQ-C30)) (Breast-23 (EORTC-QLQ-BR23))* Anxiety and depression *(Chinese version of* *the Hospital Anxiety and Depression Scale (HADS))* Body composition *(weight, waist, circumference, muscle mass, bone mineral mass, and body fat)* Drug compliance *(adherence rate)* Satisfaction *(mHealth Satisfaction Questionnaire version 2)*	16 weeks	While *physical activity intensity showed no significant changes* from baseline to week 16 (*p* = 0.24), *cognitive function* (*p* = 0.021), *future perspective* (*p* = 0.044), *arm symptoms* (*p* = 0.042), *depression* (*p* = 0.01), and *anxiety* (*p* = 0.004) *improved*. All participants reported perfect medication compliance (100%). Satisfaction with the app was high.	Education and self-management
[81]	Kim et al. (2025a) *South Korea*	Nonrandomized intervention trial	*n* = 66 Breast cancer	Cancer Manager (CAMA) app *n* = 34 Mobile health (mHealth) application	Treatment as usual *n* = 32	Self-efficacy *(10-item Korean version of the Cancer Survivor Self-Efficacy Scale (CS-SES-K))* Mental adjustment to cancer *(29-item Korean version of the Mini-Mental Adjustment to Cancer (K-Mini-MAC-Scale))* Quality of life *(World Health Organization Quality of Life Brief Version (WHOQOL-BREF))* Depression and depressive symptom severity *(9-item Patient Health Questionnaire-9 (PHQ-9))* Anxiety and anxiety symptoms *(7-item Generalized Anxiety Disorder-7 (GAD-7))* Emotional symptoms experienced by menopausal women *(Menopause Emotional Symptoms Questionnaire (MESQ))* Satisfaction *(17-item-satisfaction questionnaire based on questionnaires used in previous studies)*	nn	Throughout the intervention period, the CAMA group (vs. treatment as usual group) *demonstrated significant GAD-7* (*r* = 0.66; *p* < 0.001) and *MESQ* (*r* = 0.35; *p* =.04) *scores*. The user satisfaction survey offered insights into the CAMA app’s positive impact; trust-building outcomes; and opportunities for enhancement, such as the inclusion of communication tools and continued content enrichment. *Improvements* in the seeking help and support subscale of the Korean version of the Cancer Survivor Self-Efficacy Scale (*F*1,64 = 5.09; *p* = 0.03), the *psychological well-being* subscale of the World Health Organization Quality of Life Brief Version (*F*1,64 = 5.48; *p* = 0.02), the *anxious preoccupation* subscale (*F*1,64 = 5.49; *p* = 0.02) and *positive attitude* subscale (*F*1,64 = 5.44; *p* = 0.02) of the *K-Mini-MAC Scale*, *PHQ-9* (*F*1,64 = 4.83; *p* = 0.03), *GAD-7 *(*F*1,64 = 5.48; *p* = 0.02), and *MESQ *(*F*1,64 = 4.30; *p* = 0.04).	Education and self-management
[82]	McGeagh et al. (2023) *UK*	Feasibility study	*n* = 51 Breast cancer	HT&Me support package including animation, web-app, consultations, and email reminders Mobile health (mHealth) application	None	Feasibility and adherence measures (recruitment and retention the target population) (acceptability of the intervention) (feasibility of delivering the intervention) (research process acceptability)	Baseline, 8 weeks	HT&Me was *demonstrated as feasible to deliver*. Overall, 69% (*n* = 35) engaged with the web app; 87% (*n* = 40/46) found HT&Me helpful; and *80% (n = *36/45*) reported it motivated them to keep taking endocrine therapy*. Both consultation formats were considered acceptable. Completion of outcome measures was high. Health professionals considered HT&Me addresses an important unmet need.	Health monitoring and symptom tracking
[83]	Nápoles et al. (2019) *USA*	Single-arm feasibility study with mixed methods	*n* = 23 Breast cancer	Nuevo Amanecer survivorship care planning program: bilingual survivorship care plan, mobile app with activity tracker, survivorship booklet, and 5 telephone coaching calls Mobile health application (app)	None	Primary outcomes Self-efficacy *(new 8-item self-efficacy for managing cancer cares scale)* Symptoms *(Patient-Reported Outcomes measurement Information System (PROMIS)) (Cancer-Fatigue Scale (adapted))* Distress *(Medical Outcomes Study Health Distress Scale (Selection of 4 items))* Knowledge of cancer survivorship care *(2 global single item measures and 16-item scale)* Secondary outcomes *Emotional well-being (6-item Emotional Well-Being Scale)* Depressive and somatic symptoms *(Patient Health Questionnaire 8-item) (6-item Brief Symptom Inventory Somatization Scale)*	2 months	Compared with baseline, postintervention *fatigue* (*B* = −0.26; *p* = 0.02; Cohen *d* = 0.4) and *health distress* levels (*B* = −0.36; *p* = 0.01; Cohen *d* = 0.3) *were significantly lower* and *knowledge of recommended follow-up care and resources* (*B* = 0.41; *p* = 0.03; Cohen *d* = 0.5) and *emotional well-being improved significantly* (*B* = 1.42; *p* = 0.02; Cohen *d* = 0.3); *self-efficacy for managing cancer follow-up care did not change*. Average *daily steps increased significantly* from 6157 to 7469 (*B* = 1311.8; *p* = 0.02; Cohen *d* = 0.5).	Education and self-management
[84]	Park et al. (2022) *South Korea*	Quasiexperimental pretest–posttest design	*n* = 74 Breast cancer	12-week mobile health coaching program *n* = 37 Telehealth services	Control group *n* = 37	Symptom experience *(Memorial Symptom Assessment Scale-Short Form (MSAS-SF))* Self-management *(Korean version of the partners in health (PIH) questionnaire)* Quality of life *(Functional Assessment of Cancer Therapy-Breast (FACT-B))*	12 weeks	The intervention group showed a *significant decrease in symptom experience*, from 1.57 ± 0.46 (T0) to 1.03 ± 0.46 (T1) (*p* = 0.006), and a *decrease in psychological symptoms* from 1.71 ± 0.93 (T0) to 1.66 ± 0.69 (T2) (*p* = 0.049). *Self-management scores significantly increased* from 74.43 ± 10.72 (T0) to 76.90 ± 11.99 (T2) (*p* = 0.028). *QOL improved* from 95.83 ± 18.62 (T0) to 96.40 ± 15.35 (T2) (*p* = 0.015), and *emotional well-being increased* from 17.42 ± 4.91 (T0) to 17.50 ± 3.63 (T2) (*p* < 0.001), with all showing significant group × time interactions.	Education and self-management
[85]	Scaturro et al. (2023) *Italy*	Case–control observational study	*n* = 56 Breast cancer	Remote rehabilitation project program *n* = 24 Telehealth services	Rehabilitation project program, on an outpatient basis *n* = 32	Pain *(Numeric Rating Scale (NRS))* Upper limb disability *(Disabilities of the Arm, Shoulder and Hand questionnaire (Quick-DASH))* Fatigue *(Piper Fatigue Scale (PFS))* Quality of life *(Breast Cancer Therapy Functional Rating Scale (FACT-B))*	Baseline, 8 weeks	CG showed greater improvements than the TG in *upper limb function* (7.8 ± 4.2 vs. 10.9 ± 4.9; *p* < 0.05) and *quality of life* (27.9 ± 7.2 vs. 40.0 ± 3.3; *p* < 0.05). No difference in efficacy between the two groups was observed for pain (2.2 ± 0.6 vs. 2.3 ± 0.9; *p* = 0.64) and fatigue (3.2 ± 1.1 vs. 3.2 ± 0.6; *p* = 0.66).	Physical activity and exercise
[86]	Smith et al. (2023) *USA*	Prospective feasibility study	*n* = 250 Breast cancer	Smartphone-based ePRO symptom monitoring system Mobile health application (app)	None	Patient-reported outcomes *(Measurement Information System (PROMIS) Version 1.0)* National Cancer Institute Patient-reported outcomes version of the common terminology* (Criteria for Adverse Events (PRO-CTCAE))*	Baseline, 1 month, 3 months, 6 months, and 12 months	Primary objective was to evaluate feasibility, assessed by survey completion rates, with targets of > 65% for the baseline survey and ≥ 1 follow-up survey during the first 6 months. Secondary objectives included 12-month ET discontinuation rate (target: ≤ 15%), describing symptoms and evaluating pathway implementation. Among 250 participants, 73.2% completed the baseline survey and 69.6% completed ≥ 1 follow-up survey during the first 6 months. Thirty-one percent of participants had ≥ 1 symptom alert at baseline and 74% had ≥ 1 symptom alert during follow-up. The proportions of participants for whom pathway concordant symptom management was documented at each time point ranged from 12.8 to 36.6%. Twenty-eight participants (11.2%) discontinued ET by 12 months.	Health monitoring and symptom tracking
[87]	Smith et al. (2024) *Australia*	Single-arm, nonblinded pilot trial	*n* = 54 Breast cancer	iConquerFear was derived from the face-to-face conquer-fear intervention, which targets unhelpful beliefs about worry (i.e. metacognitions) five-module self-guided digital FCR intervention Web-based platform	None	Primary outcomes Fear of cancer recurrence* (Fear of Cancer Recurrence Inventory-Short Form (FCRI-SF))* Engagement *(total time spent using iConquerFear)* Secondary outcomes Anxiety (*Generalized Anxiety Disorder-7 (GAD-7))* Intrusive thoughts* (Impact of Event Scale-Revised (IES-R))* Negative metacognitions (*Metacognitions Questionnaire (MCQ-30))* Depression *(Patient Health Questionnaire (PHQ-9))*	Baseline, 3 months	Thirty-nine (83%) participants recorded moderate (*n* = 24; 120–599 min) or high (*n* = 15; ≥ 600 min) usage. Engagement levels increased with participant age (*p* = 0.043) but were lower in participants with higher baseline FCR (*p* = 0.028). Qualitative feedback indicated engagement was sometimes limited by difficulties with navigation and relating to featured survivors. Participants reported *significantly improved FCR* (mean reduction (95% CI): baseline to post-intervention − 3.44 (− 5.18, − 1.71), baseline to 3-month follow-up − 4.52 (− 6.25, − 2.78), *p* = < 0.001).	Psychosocial support and mental health
[88]	Williams et al. (2023) *Australia*	Feasibility study	*n* = 19 Mixed cancer types (population included 10.5% breast cancer)	Virtual stepped-care CBT program including self-help workbook (Step 1) and telehealth CBT group (Step 2) Telehealth services	None	Primary outcome Feasibility *(4-item self-report (Acceptability of Intervention Measure, Intervention Appropriateness Measure and Feasibility of Intervention measure, Client Satisfaction Questionnaire))* Secondary outcomes Fatigue *(Functional Assessment of Chronic Illness Therapy—fatigue subscale (FACIT-F))* Quality of life* (EuroQual 5 Dimension 5 Level Scale) (Perceived Self-Efficacy for Fatigue (Self-Management* *Scale))* *(Study-specific questionnaire about perceived changes in personal, social and lifestyle factors that are targeted in the REFRESH program)*	6 weeks	Step 1 Overall, participants perceived the self-help program to be feasible, appropriate, and acceptable with high ratings of program satisfaction. Step 2 Overall, participants perceived the CBT group was feasible, appropriate, and acceptable with high ratings of program satisfaction. Steps 1 & 2 *Fatigue significant overall improvement* in fatigue following Step 1 [*t*(16) = 3.7, *p* < 0.002, 95% CI (2.7, 9.7)], with a medium effect size 20 (*d* = 0.63). Self-efficacy for managing fatigue. *Significant improvement in self-efficacy* in managing fatigue following completion of Step 1 [*t*(16) = 3.8), *p* < 0.001, 95% CI (0.7, 2.3)] with medium effect size 20 (*d* = 0.7). Quality of life. Quality of life scores improved following Step 1; however, this *result did not reach statistical significance* (*t*(16) = − 1.5, *p* = 0.14). Perceived changes in symptoms and activity. After Step 1, almost half the participants *perceived improvements in mood, exercise levels, social activities, hobbies, and/or concentration/motivation.*	Psychosocial support and mental health
[89]	Wolff et al. (2023) *Germany*	Feasibility pilot study	*n* = 60 Breast cancer	App-based lifestyle coaching (PINK!) (nutrition, physical activity, and mental health) *n* = 38 Mobile health (mHealth) application	Waiting list standard care *n* = 22	Primary outcome Psychological distress (Patient Health Questionnaire (PHQ-9)) Secondary outcomes Quality of life *(European Organization for the Research and Treatment of Cancer Quality of Life Questionnaire (EORTC-QLQ-C30))* Physical activity level *(International Physical Activity Questionnaire (IPAQ))*	12 weeks	Primary efficacy variable analysis revealed a relative average *decrease* of 32.9% in *psychological distress*, which corresponds to a statistically significant reduction (*p* < 0.001) within 12 weeks compared to the control group. Linear regressions within usage groups showed a correlation of high app usage and a reduction of psychological distress. *Fatigue* data *presented a statistically significant antifatigue efficacy* (*p* < 0.001) and physical activity increased by 63.9%.	Education and self-management
[90]	Clara et al. (2025) *Portugal*	Mixed-methods study	*n* = 123 Mixed cancer types (population included 76.4% breast cancer, 5.6% gynecological cancer)	Digital cognitive-behavioral therapy for insomnia (CBT-I) program OncoSleep Mobile health (mHealth) application	None	Outcome variables and measures Acceptability Usability *(User Experience Questionnaire-Short (UEQ-S))* *(acceptability questionnaire, qualitative thematic analysis of participant feedback)* Satisfaction *(Consumer Report Treatment Satisfaction Scale) (Treatment Satisfaction Scale)* Sleep quality* (Insomnia Severity Index (ISI))*	Not reported	The digital CBT-I, OncoSleep, received high ratings for perceived efficacy, satisfaction, helpfulness, usability, likelihood of future use of therapeutic techniques, likelihood of recommendation, and user experience. Patients who showed clinically significant improvements in insomnia severity reported better user experience.	Psychosocial support and mental health
[91]	Gitonga et al. (2025) *Ireland*	Cross-sectional survey	*n* = 44 (total) Mixed cancer types (population included 76% breast cancer)	Online platform Cancer Thriving and Surviving (CTS) program, evidence-based, self-management program designed to empower cancer patients transitioning from active treatment to survivorship Telehealth services	None	Usability *(Telehealth Usability* *Questionnaire (TUQ))* Usefulness of CTS sessions Overall impact of participation* (motivations, supports and satisfaction. psychological well-being, QoL and empowerment)*		Motivations for engaging in CTS included seeking peer support, psychosocial assistance, and practical self-management tools. Most respondents *agreed that the program improved their psychological wellbeing* (90%) and *quality of life* (76%) and helped them take *more control of their health* (83%). TUQ scores indicated *high usability* of the CH systems.	Education and self-management
[92]	Mohammadzadeh (2022) *Iran*	Pre–post methodological study, descriptive cross-sectional study	First phase *n* = 120 Second phase *n* = 24 Third phase *n* = 24 Breast cancer	Mobile app providing educational content and self-management tools First phase Identifying educational content, designing the user experience Second phase Developing and implementing Third phase Pre- and post-test Mobile health (mHealth) application	Pre-intervention baseline	Third phase *(Quality of Life in Adult Cancer Survivors questionnaire (QLACS))*	3 months	According to the results of the pre- and post-implementations among the most significant changes were in the quality-of-life level, highest respectively: social avoidance (pre: 6.41–post: 3.56), negative feelings (pre: 5.93–post: 3.40), sexual function (pre: 6.80–post: 5.04), sexual interest (pre: 6.41–post: 4.75), and pain (pre: 6.37–post: 4.97). And the least changes respectively: distress-family (pre: 7–post: 7), distress-recurrence (pre: 4.49–post: 4.38), benefits (pre: 2.47–post: 3.12), appearance (pre: 4.10–post: 3.32).	Education and self-management

### Risk of bias

Different risk-of-bias tools were applied depending on study design. Randomized controlled trials were assessed using the Cochrane Risk of Bias 2 (RoB2) tool [93]. Nonrandomized studies were evaluated using the ROBINS-I tool, ensuring appropriate assessment across study designs [94]. Additional tools, including the Joanna Briggs Institute (JBI) checklist and the Mixed Methods Appraisal Tool (MMAT), were applied where appropriate [95, 96]. The risk of bias of the included studies was assessed independently by two reviewers (NK, LLM).

The ratings were compared, and disagreements in the assessment were further discussed among the research team members to reach a consensus.

### Data synthesis

Data synthesis focused on the effectiveness of interventions in relation to the outcomes. Extracted data were reported in line with the PRISMA checklist. Data were synthesized narratively. Due to the expected high level of heterogeneity of the included studies in terms of design, comparators, and outcome measures, a meta-analysis was not conducted. Effect sizes were reported where available and interpreted descriptively due to heterogeneity. Data were organized and structured to describe patterns across the results. All results were considered and reported in relation to overall study quality.

## Results

### Study selection and characteristics

**Fig. 1:**
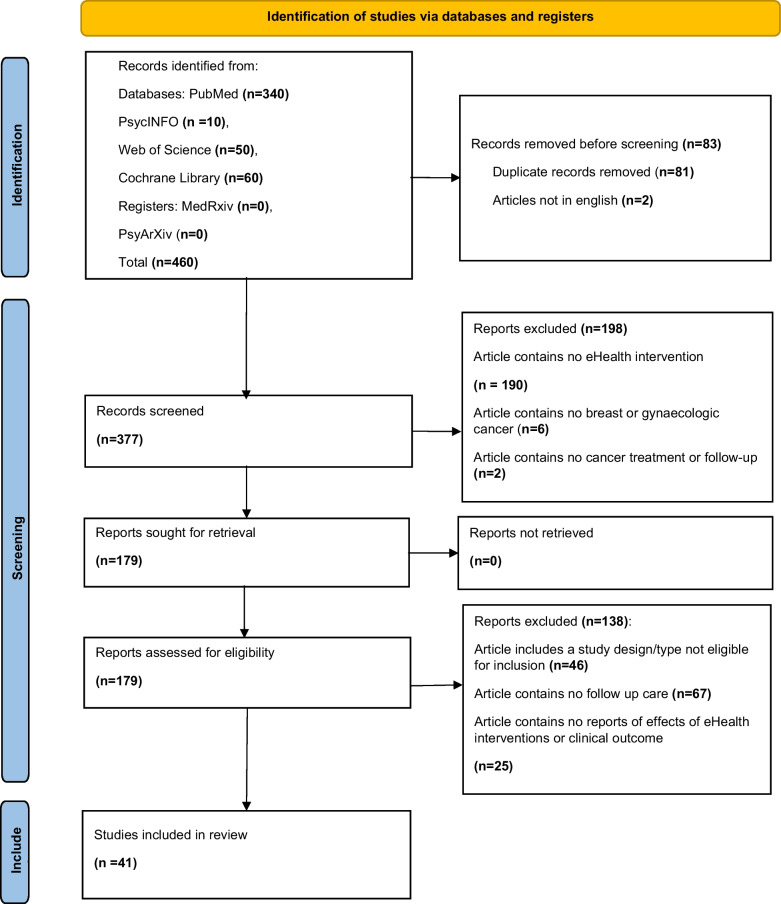
Flow diagram of the literature search and filtering results for a systematic review of the effects of eHealth interventions used in cancer follow-up care for breast cancer and gynecological oncologic patients

Initial searches of the six databases (PubMed, PsycINFO, Web of Science, Cochrane Library, MedRxiv, and PsyArXiv) returned 460 records. After removal of duplicates and ineligible records (*n* = 83), 198 were excluded during title and abstract screening, and a further 138 were excluded following full-text review. A total of 41 studies met the inclusion criteria (Fig. 1).

The studies were published between January 2010 and February 2026 and included 26 randomized controlled trials (RCTs) [52–77], 12 nonrandomized control trials (non-RCT) [78–89], and other designs [90–92]. The studies were conducted in multiple countries.

### Sample size and study population

Sample sizes varied considerably, ranging from 19 participants [88] to 2712 [65]. While some larger studies included between 350 and over 600 participants [71, 73, 74], most studies had moderate sample sizes, typically between 50 and 200 participants.

The evidence base was predominantly focused on breast cancer survivors (*n* = 29) [52, 56–64, 67–70, 72, 73, 76, 78, 79, 87, 89, 92], with fewer studies including mixed cancer populations (including breast and gynecological cancer survivors) (*n* = 11) [53–55, 65, 66, 71, 74, 77, 88, 90, 91], and only one study exclusively involving gynecological cancer survivors (ovarian cancer) [75]. Additionally, two studies focused on specific subgroups of breast cancer survivors, namely, Spanish-speaking American women [69] and Asian American women [62].

### Comparator

Most studies used usual care or no intervention as a comparator. Usual care generally consisted of routine follow-up by oncologists or primary care providers, with limited emphasis on post-treatment symptom management. A small number compared eHealth interventions with face-to-face approaches or alternative digital interventions (Table 1).

### Follow-up period

Follow-up periods varied from 2 weeks [66] to 36 months [61], with most studies reporting short-term outcomes (≤ 16 weeks). Only a small number of studies included follow-up beyond 12 months [54, 65, 86], and even fewer extended follow-up to 18 months or longer [61, 70].

### Risk of bias

Among the randomized controlled trials (RCTs) assessed using the RoB 2 tool, the majority were judged to have a low risk of bias (*n* = 16) [53, 54, 56, 58, 60, 62, 63, 66, 67, 70–72, 74, 76, 77], indicating generally robust methodological quality. However, several studies raised concerns, with six studies [55, 61, 65, 69, 73, 75] rated as having “some concerns” and four studies [52, 57, 64, 68] classified as having a high risk of bias.

In contrast, nonrandomized studies evaluated using the ROBINS-I tool predominantly exhibited higher levels of bias. Of these, nine studies [79–85, 87, 88] were judged to have a serious risk of bias, while three [78, 86, 89] showed a moderate risk.

Studies assessed with alternative appraisal tools showed mixed results. Cross-sectional and pre–post studies evaluated using the JBI Critical Appraisal Checklist were generally rated as having a moderate risk of bias [91, 92], while the single mixed-methods study assessed using the MMAT demonstrated a low risk of bias [90].

Overall, although a substantial proportion of RCTs demonstrated high methodological quality, the overall evidence base is limited by the moderate to serious risk of bias observed in nonrandomized studies. This heterogeneity in study quality will be considered when interpreting the findings of this review (Table 2.).

**Table 2.**
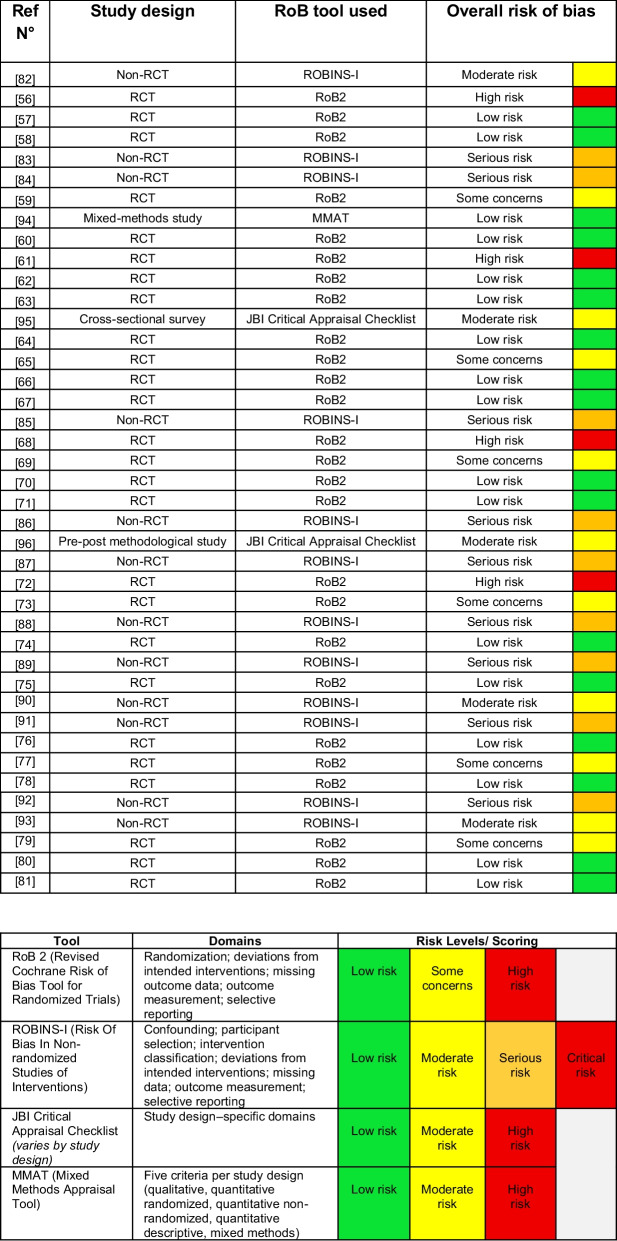
Risk of bias assessment of included studies and appraisal tools used

### eHealth intervention: delivery mode

The eHealth interventions were delivered in a variety of ways. Delivery modes were predominantly as follows:

- Mobile health (mHealth) application [52–55, 57, 60, 64, 66–68, 72–74, 77–83, 86, 89, 90, 92].
- Telehealth services [58, 59, 61, 63, 69–71, 84, 85, 88, 91].
- Web-based platforms [62, 65, 75, 76, 87].
- Virtual reality (VR) and interactive tools [56].

### eHealth intervention: content

**Fig. 2:**
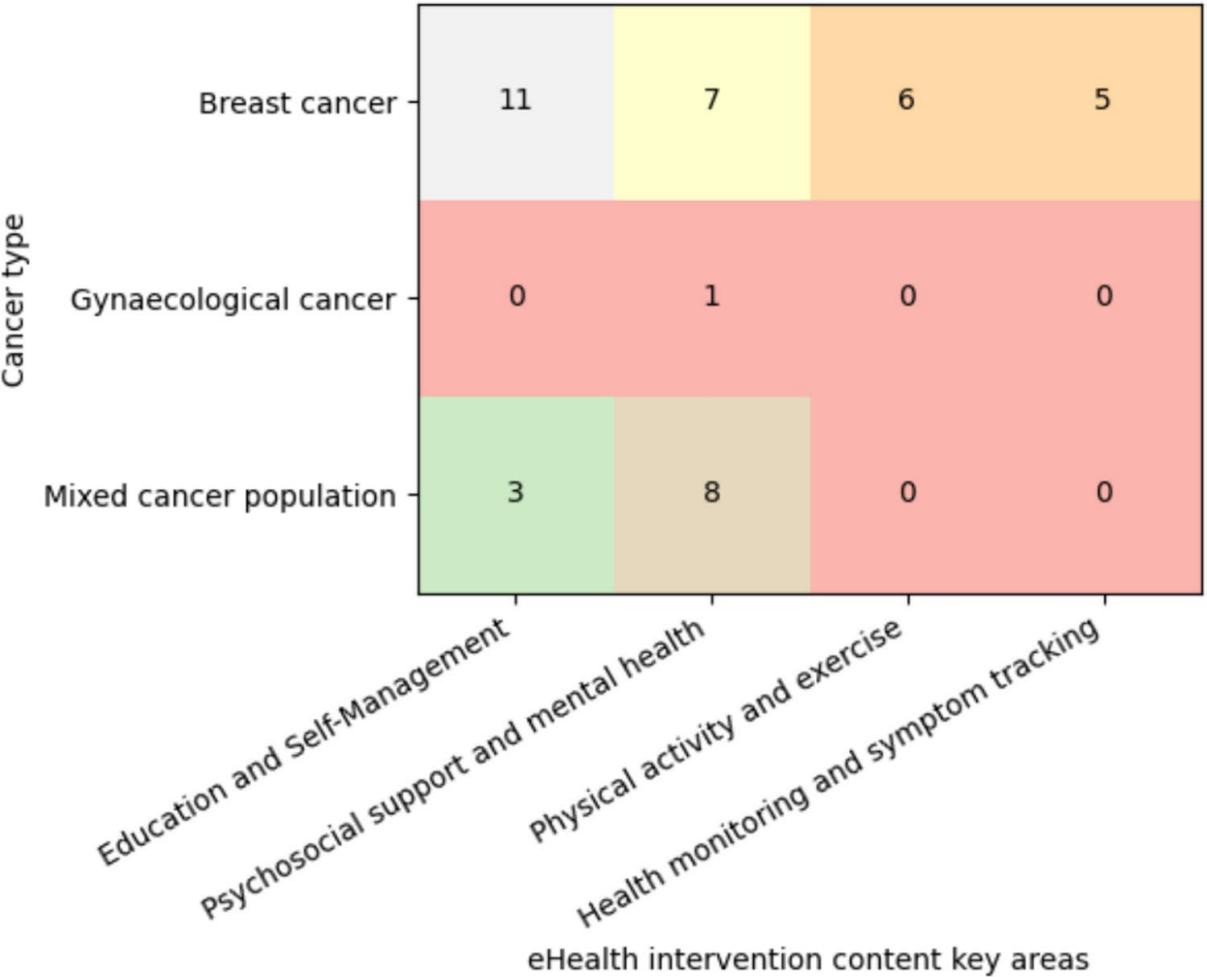
Evidence map of included studies according to eHealth intervention content key areas and cancer type. Numbers within cells indicate the number of studies addressing each intervention content area within the respective cancer population

An evidence map was developed to visualize the distribution of included studies across eHealth intervention content key areas and cancer types (Fig. 2).

Intervention content clustered into four key areas:

1. *Education and self-manageme*nt: Most identified interventions focused on education and self-management, representing the most frequently addressed content area across studies [52, 61, 62, 64, 65, 74, 78, 80, 81, 83, 84, 89, 91, 92].
2. *Physical activity and exercise:* A smaller number of studies addressed physical activity and exercise interventions, including digital rehabilitation programs, remote exercise coaching, and lifestyle modification tools [56, 59, 70, 72, 79, 85].
3. *Psychosocial support and mental health:* Another prominent intervention domain involved psychosocial support and mental health, including digital stress-management programs, mindfulness-based interventions, and cognitive behavioral therapy–based applications [53–55, 57, 58, 66, 67, 69, 71, 73, 75–77, 87, 88, 90].
4. *Health monitoring and symptom tracking:* In contrast, health monitoring and symptom tracking interventions represented a smaller but emerging category [60, 63, 68, 82, 86].

Overall, the evidence map indicates that current eHealth interventions in cancer follow-up care primarily target self-management support and psychosocial well-being. At the same time, the limited representation of gynecological cancer populations underscores a significant research gap.

### Measurement tools and outcomes

The effects of eHealth interventions on breast and gynecological cancer survivors were evaluated across multiple outcome domains, with individual studies contributing to more than one category (Table 1). The most commonly assessed outcomes included quality of life (QoL), symptom burden, mental health, fatigue, symptom management, and physical activity.

*Quality of life (QoL)* was measured using instruments such as the EORTC QLQ-C30 (European Organization for the Research and Treatment of Cancer Quality of Life Questionnaire), the FACT-G/B (Functional Assessment of Cancer Therapy-General/Therapy-Breast), and the SF-36 (Medical Outcomes Study 36-item short form survey) [53–55, 58, 59, 62, 63, 65, 68, 70, 71, 73, 74, 78, 80, 81, 84, 85, 88, 89].

*Burden of treatment* was measured using instruments such as Edmonton Symptom Assessment Scale (ESAS), Memorial Symptom Assessment Scale–Short Form (MSAS-SF), Patient-Reported Outcomes version of the Common Terminology Criteria for Adverse Events (PRO-CTCAE), Functional Assessment of Cancer Therapy-Endocrine Symptoms (FACT-ES) [60, 62, 63, 66, 70, 78, 84].

*Mental health* outcomes, including anxiety, depression, stress, and distress, were measured using validated instruments such as the Hospital Anxiety and Depression Scale (HADS), the Patient Health Questionnaire (PHQ-2/9), the Perceived Stress Scale (PSS-14), the Generalized Anxiety Disorder-7 Scale (GAD-7), and PROMIS measures including the PROMIS-Depression Short Form (PROMIS-D) and PROMIS-Anxiety Short Form (PROMIS-A) [53, 54, 56, 57, 63, 65–67, 73, 77, 79–81, 87].

*Fatigue* was measured using scales such as the Piper Fatigue Scale and Piper Fatigue Scale–Revised (PFS/R-PFS), the Functional Assessment of Chronic Illness Therapy–Fatigue (FACIT-F), the Cancer Fatigue Scale (CFS), the Visual Analogue Scale (VAS), and the Self-Regulatory Fatigue Scale (SRF-18) (Self-Regulatory Fatigue18) [52, 54, 56, 57, 67, 70, 73, 76, 79, 85].

*Symptom management* was measured using a variety of instruments. Sleep disturbance was measured using the Insomnia Severity Index (ISI) and the Pittsburgh Sleep Quality Index (PSQI); pain was measured using the Numeric Rating Scale (NRS) and the Brief Pain Inventory (BPI); and medication adherence was assessed using the Morisky Medication Adherence Scale (MMAS-4) and the Medication Adherence Report Scale (MARS5) [57–60, 63, 66–69, 73, 76, 90].

*Physical activity and fitness* were assessed using both self-report and objective measures, including the International Physical Activity Questionnaire (IPAQ), moderate-to-vigorous physical activity (MVPA), the 6-min walk test (6MWT), the Activation-Deactivation Adjective Check List (AD ACL), and the Godin-Shephard Leisure-Time Physical Activity Questionnaire (GSLTPAQ) [56, 67, 72, 79, 80, 89].

Although infections, malnutrition, and unplanned hospital readmissions were initially defined as outcomes of interest, none of the included studies explicitly assessed these outcomes. Therefore, these outcomes were not included in the final synthesis.

### Effectiveness of eHealth interventions

#### Breast cancer survivors

Across the included studies, eHealth interventions for breast cancer survivors were associated with improvements across multiple domains, including fatigue, psychological outcomes, cognitive function, sleep quality, and physical well-being [56, 67, 76]. However, the magnitude of these effects varied systematically with methodological quality.

Studies with a higher risk of bias consistently reported large effect sizes across outcomes, often exceeding *d* = 0.9. For example, substantial reductions in fatigue alongside improvements in anxiety and depression were reported for an aerobic exercise and technology-guided mindfulness training [56].

Similarly, another study demonstrated large effects on cognitive performance (TMT-A *d* = 1.1–1.2), psychological state (HADS *d* = 1.6), and functional capacity (*d* = 1.5), with additional benefits for fatigue and manageability using a mHealth app for an 8-week occupational therapy program [67]. Another trial reported moderate to large effects on sleep quality and fatigue during an internet-delivered Cognitive-Behavioral Therapy (CBT-I), with post-treatment effect sizes ranging from *d* = 0.33 to 1.17 and follow-up effects from *d* = 0.66 to 1.10, suggesting sustained improvements over time [76].

In contrast, studies with lower or moderate risk of bias showed smaller but more consistent effects, typically in the small to moderate range [70, 83]. A trail reported improvements in fatigue (*d* = 0.4), health distress (*d* = 0.3), emotional well-being (*d* = 0.3), and knowledge (*d* = 0.5), alongside gains in self-efficacy and reductions in depressive symptoms [83]. Similarly, another study found small to moderate effects on physical quality of life (*d* = 0.40), musculoskeletal pain (*d* = 0.49), and body image (*d* = 0.31), while also addressing fatigue and menopausal symptoms [70].

#### Gynecological cancer survivors

Evidence for gynecological cancer was limited to a single study with some concerns regarding risk of bias [75]. No significant between-group effects were observed for key psychological outcomes, including fear of cancer recurrence (FCR) and fear of progression (FoP) [75].

#### Mixed cancer populations

Studies including mixed cancer populations including breast and gynecological cancer survivors showed heterogeneous effects across psychosocial and symptom-related outcomes, with effect sizes according to methodological quality [55, 66, 77].

A clear pattern emerged in which larger effects were predominantly reported in studies with higher risk of bias, such as substantial improvements in physical well-being, sense of coherence, and cancer-related distress [55]. In contrast, studies with more robust designs demonstrated small and inconsistent effects, including modest reductions in anxiety and depression [77] or negligible between-group differences with potential nonspecific effects, such as improvements observed in control groups [66].

Overall, the evidence suggests that eHealth interventions may improve psychological and coping-related outcomes, but effect sizes are inconsistent and likely influenced by study quality.

## Discussion

### Comparison with existing evidence

The present findings align with a growing body of literature suggesting that eHealth interventions in cancer survivorship are associated with heterogeneous and generally modest effects. Several systematic reviews have highlighted the limited robustness of the evidence base despite the rapid expansion of digital interventions. While numerous mobile applications for breast cancer care exist, the current evidence base for their effectiveness is still evolving and, in many cases, relies on studies with methodological limitations [97]. Similarly, findings on mental health outcomes have been mixed; however, this variability reflects the diversity of intervention designs and study approaches, highlighting opportunities for further high-quality research to strengthen conclusions [98]. Consistent with these findings, randomized controlled trials suggest that interventions can lead to small to moderate improvements in physical activity and dietary behavior; however, results vary across studies, indicating potential for further optimization to enhance effectiveness [99]. Likewise, mHealth applications show promise in enhancing quality of life; however, the current evidence is diverse and reflects varying study designs, underscoring the need for further robust research to confirm and extend these benefits [100].

Importantly, this pattern is reinforced by broader developments in the field. Reviews of personalized follow-up care indicate that even structured and tailored interventions yield variable and often modest benefits, underscoring persistent challenges in demonstrating clear clinical effectiveness [101]. Concurrently, development studies reflect the increasing sophistication and user-centered design of mHealth interventions, underscoring a persistent gap between technological innovation and the availability of robust evidence of effectiveness [102].

The current evidence base is heavily dominated by studies involving breast cancer survivors, whereas evidence for gynecological cancer populations remains limited. This imbalance may reflect differences in research priorities, funding allocation, and recruitment feasibility, but it raises important concerns regarding the generalizability of findings. Given the distinct symptom burden, treatment sequelae, and survivorship needs of gynecological cancer patients, it remains unclear whether the observed effects of eHealth interventions can be directly transferred to this population. This gap highlights the need for more targeted research addressing the specific requirements of gynecological cancer survivors.

### Interpretation of methodological patterns

This review synthesized evidence on the effectiveness of eHealth interventions in breast and gynecological cancer survivors and identified inconsistent and generally modest effects (Table 3). A key finding was that studies with higher risk of bias consistently reported larger and more favorable effects, whereas methodologically robust trials showed smaller or nonsignificant effects [8, 53, 54, 64, 66, 67, 77, 81, 83, 84, 87,88 vs. ]. These findings suggest that eHealth interventions can yield meaningful benefits across multiple outcomes, with effect sizes that are likely moderate overall; at the same time, they under score the importance of rigorous study designs to ensure that these effects are estimated as accurately and reliably as possible.

**Table 3:** Key outcomes measured and effect sizes stratified by cancer type

Study	Key outcomes measured	Effect size
Breast cancer		
[78]	General health, quality of life, symptoms	Adjusted mean differences: general health − 1.55; general QoL − 2.39; specific QoL − 4.31; symptom change not significant, NR*
[52]	Fatigue, body image	Significant improvement in fatigue and body image; NR*
[80]	Feasibility, adherence, physical activity, health-related quality of life, anxiety, depression, body composition, drug compliance, satisfaction	Significant improvements in several psychosocial outcomes; NR*
[56]	Feasibility and acceptance, fatigue, tiredness and energy, anxiety and depression	**Cohen’s *d* = 0.91** for fatigue reduction favoring combined intervention vs. exercise only
[57]	Anxiety, depression, fatigue, insomnia, cancer-related worry, vasomotor symptoms, mindfulness and awareness	Mean differences: anxiety − **4.13**; depression − **6.03**; fatigue + **6.03**; insomnia − **3.97**; cancer worry − 4.57, NR*
[58]	General HRQoL, breast cancer–specific QoL, perceived cognitive function, spiritual well-being, distress, sleep disturbances	Clinically significant improvements reported; NR*
[59]	Quality of life, pain, isometric strength, fatigue	Significant group differences favoring telerehabilitation; NR*
[60]	Aromatase inhibitor (AI) adherence, symptom burden	Weekly use **74% vs. 38%**; adherence at 8 weeks **100% vs. 72%**; symptom burden trend favored reminders; NR*
[61]	Work ability	Between-group mean differences close to zero (**− 0.21 to 0.48**); no additional effect vs. control, NR*
[62]	Supportive care needs, symptom distress, quality of life	Significant improvement in QoL; symptom reductions not statistically significant; standardized ES NR*
[63]	Satisfaction, medication adherence, therapy satisfaction, quality of life, anxiety and depression, stress coping, self-efficacy	Symptom distress ***B* = − 1.91;** better self-management reported; NR*
[81]	Self-efficacy, mental adjustment to cancer, quality of life, depression, anxiety, emotional symptoms, satisfaction	Significant improvements across outcomes; *F*-statistics reported, NR*
[64]	Self-efficacy, health behavior, eating habits, physical activity, cardiometabolic risk factors	Significant increase in vegetable intake (*p* = 0.017) and decrease in fasting glucose (*p* = 0.037); NR*
[67]	Cognitive performance, psychological state, pain, fatigue, functional capacity	TMT-A *d* = 1.1–1.2; anxiety/general HADS *d* = 1.6; functional capacity *d* = 1.5; manageability and fatigue also improved
[82]	Feasibility and adherence	Feasibility/acceptability study; NR*
[92]	Quality of life	Large pre–post improvements in several QoL domains; SUS 83/100; NR*
[83]	Self-efficacy, symptoms, distress, knowledge, depression	Fatigue *B* = − 0.26, *d* = 0.4; health distress *B* = − 0.36, *d* = 0.3; knowledge *B* =.41, *d* = 0.5; emotional well-being *B* = 1.42, *d* = 0.3
[68]	Health-related quality of life, communication, patient-reported outcome, mediation adherence	FACT-B difference − 1.55; no significant QoL effect, NR*
[69]	Acceptability and feasibility, sleep quality	Pilot RCT reported improvement; NR*
[84]	Symptom experience, self-management, quality of life	Significant group × time improvements; NR*
[70]	Weight, body composition, quality of life, fatigue, arthralgia, menopausal symptom, fear of cancer recurrence, body image, adverse events	Physical QoL *d* = 0.40; musculoskeletal pain *d* = 0.49; body image *d* = 0.31
[69]	Pain, upper limb disability, fatigue, quality of life	Outpatient rehabilitation superior on some outcomes; NR*
[87]	Fear of cancer recurrence, engagement, anxiety, intrusive thoughts, negative metacognitions, depression	Mean FCR reduction − 3.44 post and − 4.52 at 3 months; NR*
[86]	Patient-reported outcomes	Feasibility targets met; 12-month ET discontinuation 11.2%; NR*
[72]	Physical activity, patient-reported outcomes	Significant overall PRO improvement; no significant component-specific differences; NR*
[73]	Perceived stress, anxiety, depression, fatigue, health-related quality of life, mindfulness, coping, sleep, app use	No significant differences vs. usual care; stress MDs − 0.28 and − 0.42 NR*
[89]	Psychological distress, quality of life, physical activity level	Psychological distress reduced 32.9%; physical activity increased 63.9%; NR*,
[76]	Sleep quality, fatigue	Post-treatment *d* = 0.33–1.17; follow-up *d* = 0.66–1.10
[79]	Weight gain/loss, physical activity, nutrition, fatigue, depression	NR*
Gynecological cancer		
[75]	Feasibility, acceptability, safety, fear of cancer recurrence, fear of progression	No significant group differences in FCR/FoP; acceptability mean 3/4; 11% withdrew due to distress, NR*
Mixed cancer types		
[90]	Acceptability, usability, satisfaction, sleep quality	User experience, usability, acceptability study, NR*
[91]	Usability, overall impact of participation	Descriptive effectiveness: psychological well-being 90%, QoL 76%, health control 83%; NR*
[65]	Quality of life, depression, anxiety	Global health at 12 months: 3.06 and 2.78 point improvements; QoL benefit at 12 months for supported arm 1.42; cost differences also reported, NR*
[66]	Symptom burden, anxiety, depression, sleep quality	Within-group depression in control *d* = − 0.80; meditation-group anxiety change *d* = − 0.29; group differences small/limited
[71]	Health-related quality of life	Significant improvement in general health, bodily pain, vitality, and global physical/mental health; NR*
[88]	Feasibility, fatigue, quality of life	Feasibility study; NR*
[77]	Anxiety, depression	Anxiety *d* = 0.25; depression *d* = 0.24
[53]	Stress, anxiety, depression, health-related quality of life, acceptability and feasibility	Stress MD − 2.8; role physical MD 17.7; NR*
[54]	Stress, anxiety, depression, fatigue, health-related quality of life	Largest 6-month effects: stress MD − 5.1; anxiety − 1.4; depression − 2.1; self-regulatory fatigue − 4.9, NR*
[55]	Feasibility, sense of coherence, cancer-specific distress, health-related quality of life, acceptability	Physical well-being *d* = 1.04; sense of coherence *d* = 0.76; distress *d* = 1.03
[74]	Patient activation, health-related quality of life	NR*

^*^NR not reported, standardized ES refers to effect sizes such as Cohen’s *d* or Hedges’ Values in bold indicate statistically significant results.

### Sustainability of effects and clinical relevance

The findings further suggest that intervention effects may diminish over time, particularly in the absence of continued support or reinforcement. This raises important concerns regarding the durability and real-world applicability of many eHealth interventions, especially those designed as fully self-managed tools.

Reliance on short-term outcome assessments may contribute to an overestimation of effectiveness, as early engagement and novelty effects are more likely to influence initial outcomes. Moreover, a temporal mismatch between intervention goals and outcome assessment becomes apparent: Many interventions target behaviors that require long-term maintenance, yet their effectiveness is primarily evaluated over short durations.

These findings highlight that it remains unclear whether eHealth interventions produce transient improvements or sustained clinical benefits. Future research should therefore prioritize longitudinal designs with extended follow-up, incorporating maintenance phases, booster components, and repeated outcome assessments to better capture the trajectory and sustainability of intervention effects.

### Limitations

This review is subject to several limitations related to the underlying evidence base. First, this review highlights a clear imbalance in the representation of cancer types across included studies. The evidence base is heavily dominated by breast cancer populations, whereas gynecological cancers are substantially underrepresented and frequently included only as small subgroups within mixed cancer samples. The lack of dedicated studies in gynecological cancer populations restricts the ability to draw robust and cancer-specific conclusions, underscoring a critical gap in the current literature.

Second, a substantial proportion of included studies were nonrandomized and rated as having moderate to serious risk of bias [79–81, 83, 84, 87, 88]. Even among RCTs, important issues were identified, leading to “some concerns” or “high risk of bias” ratings in multiple studies [61, 68, 69, 73]. These limitations restrict the strength of conclusions that can be drawn regarding eHealth intervention effectiveness.

Third, most studies evaluated outcomes within short-term timeframes (≤ 12 weeks), with relatively few extending beyond 6 months. This limits the ability to assess the sustainability of intervention effects, particularly for outcomes that inherently require long-term maintenance, such as behavioral change, quality of life, and self-management.

Fourth, there was a lack of alignment between intervention targets and follow-up duration. Many interventions aimed to modify long-term behaviors, yet follow-up periods were often insufficient to determine whether these changes were sustained beyond the intervention phase.

Finally, heterogeneity in intervention design and outcome measures further limits comparability across studies and reduces the overall coherence of the evidence base.

### Implications for cancer survivorship

The findings of this review suggest that eHealth interventions hold promise as a supportive approach in cancer survivorship care, with beneficial effects across a range of outcomes. However, evidence for gynecological cancer survivors remains scarce, limiting the generalizability of findings and raising uncertainty about the suitability of existing interventions for this group. As survivorship care needs continue to increase, eHealth interventions may contribute to more accessible, scalable, and patient-centered models of follow-up care, potentially helping to address gaps in long-term support. Nevertheless, their long-term effectiveness and applicability across diverse cancer groups, particularly gynecological populations, remain insufficiently understood. These findings highlight the need for future research to prioritize rigorous study designs, extended follow-up periods, and the development of tailored, cancer-specific interventions in order to better meet the complex and evolving needs of cancer survivors.

### Implications for research

Future research should address the current imbalance in cancer type representation by prioritizing underrepresented populations, such as gynecological cancer survivors. In addition, eHealth interventions should be tailored to the distinct clinical and psychosocial needs of specific cancer groups rather than relying on generic approaches. There is need for methodologically rigorous studies, including adequately powered randomized controlled trials with standardized outcome measures and extended follow-up periods to evaluate the sustainability of intervention effects. Furthermore, future studies should incorporate strategies to enhance engagement and adherence—such as personalization and user-centered design—in order to improve the effectiveness and real-world applicability of eHealth interventions in survivorship care.

## Conclusions

The available evidence suggests that eHealth interventions have the potential to support cancer survivorship care. While the observed effect sizes are generally modest for breast cancer survivors, they are consistent with the complex and multifactorial needs of cancer survivors in general. Continued progress in this field will depend on rigorously conducted and transparently reported trials, as well as longer follow-up periods and broader inclusion of underrepresented populations, such as gynecological cancer survivors, to strengthen the evidence base and enhance the generalizability of findings.

## Data Availability

No datasets were generated or analyzed during the current study.
